# Biocompatible Piezoelectric PVDF/HA/AgNO_3_ Thin Film Prepared by the Solvent Casting Method

**DOI:** 10.3390/s23010289

**Published:** 2022-12-27

**Authors:** Ieva Markuniene, Marzieh Rabiei, Sohrab Nasiri, Sigita Urbaite, Arvydas Palevicius, Giedrius Janusas

**Affiliations:** Faculty of Mechanical Engineering and Design, Kaunas University of Technology, Studentu Street 56, LT-51373 Kaunas, Lithuania

**Keywords:** PVDF, HA, AgNO_3_, DMSO, thin film, X-ray diffraction

## Abstract

In this study, new composites based on polyvinylidene fluoride (PVDF) were ornamented and prepared with hydroxyapatite (HA) and silver nitride (AgNO_3_). Taking into account the polarity of the solvent dimethyl sulfoxide, this solvent was used to disperse the particles. The aim of using DMSO was to create amorphous phases and the strong dipoles of the C–F bond to reduce the energy barrier and improve the electrical properties. The PVDF played the role of matrix in HA, and AgNO_3_ was used as reinforcing elements. X-ray diffraction of the samples directly showed the amorphous phase and mixed amorphous and crystalline phases when all three materials were used simultaneously for preparing the composite. The scanning electron microscopy (SEM) images of the samples confirmed the role of PVDF, HA, and AgNO_3_. Furthermore, the energy dispersive X-ray (EDX) analysis was performed and proved that the HA structure did not change when the ratio of $\frac{\mathrm{Ca}}{P}$ was equal to the ratio of natural HA. The electrical properties were investigated, and the amount of energy ranged from 56.50 to 125.20 mV. The final results showed that a designed device consisting of an active layer made of 0.1 g HA:0.5 g PVDF showed the highest energy barrier, the highest polarity, and surface energy, thus proving its relevance as potential material for energy harvesting applications.

## 1. Introduction

Composites are formed by combining two or more materials with different properties without dissolving or mixing them into each other [1]. Most composites are made by surrounding one material (the matrix) with a stronger material (the reinforcement) [2]. Polymers generally have high dielectric degradation and high field strength. Due to these properties, piezoelectric polymers have excellent applications in technical and engineering fields, and in various device configurations [3]. Polyvinylidene fluoride (PVDF) is one of the most attractive polymer materials in the membrane industry, especially in the water treatment industry, due to its excellent stability, membrane forming, and electrical properties [4]. PVDF has a simple polymeric structure, with a repeating unit of chemical formula (C_2_H_2_F_2_)_n_ [5]_._ PVDF is a semi-crystalline polymer of the fluoropolymer family, characterized by piezoelectricity, high thermal stability, and mechanical strength [6]. PVDF can be comparable to or even greater than ferroelectric ceramics while maintaining high compliance due to its polymeric nature [7]. PVDF has three distinct crystalline phases, namely α, β, and γ, and β is the only non-polar phase and the most common of all [8]. PVDF is widely used in electrical engineering due to its good ferroelectric properties and is the most favorable among the fluoride polymers [9]. Basically, PVDF is a classical ferroelectric polymer that is partially crystalline and has the dipole moment perpendicular to the polymer chain, especially when the polar all-trans β-phase dominates [10]. PVDF is considered a promising candidate for self-powered smart electronic devices that convert weak mechanical energy into electrical signals. These features have great potential for sensor applications, energy harvesting and storage devices, and biomedical materials, especially in the field of wearable or implantable devices [11]. PVDF and its derivatives are suitable to overcome the limitations of ceramic-based piezoelectric materials, especially their brittleness [12]. To improve the hydrophilicity of PVDF membranes, various strategies have been investigated, such as physical mixing, chemical grafting, and surface modification [13,14]. Hydroxyapatite (HA) is widely used for the preparation of biological coatings on the surface of metal due to its structural and chemical similarity to natural bone minerals and its good biological activity [15]. The piezoelectric response of PVDF can be increased when the β phase of PVDF is increased; therefore, the piezoelectric property of PVDF can be improved with porous ceramics like hydroxyapatite as reinforcement [3]. HA is a versatile biomaterial with the chemical composition Ca_10_(PO_4_)_6_(OH)_2_ that has important applications in biomedical engineering such as bone scaffolds, drug delivery systems, dental implants and bone fillers, implant coatings, and chromatography (protein processing) [16]. HA is endowed with all kinds of morphologies. Among them are three-dimensional hydroxyapatite nanoparticles, which have much more hydrophilic groups, better mechanical properties, larger specific surface area, larger adsorption amount, and larger pore volume [17]. Several methods have been described for the synthesis of HA nanoparticles, including wet chemical sol-gel, hydrothermal, heat treatment, and microwave methods [18,19]. Considering that HA has good hydrophilicity and can form a bony bond, it is possible to produce a HA/PVDF coating. The introduction of HA into the piezoelectric PVDF coating can improve the hydrophilicity of the coating and gives the coating the piezoelectric properties of PVDF and the good biocompatibility of HA [20]. The addition of a conductive phase such as silver can be helpful, too. The conductive phase improves charge transfer and increases the sensitivity of the piezoelectric response [21]. Silver (Ag) is one of the inorganic antibacterial agents that have been extensively studied recently. Silver is believed to have antibacterial activity through the formation of silver ions [22]. There are some studies on PVDF/HA as control targets. For example, Hussein et al. used a PVDF/HA composite to prevent the severe corrosion of 316L stainless steel; thus, good agreements in electrochemical corrosion parameters were obtained and reported [23]. The adsorption performance of hybrid PVDF membranes by HA was investigated by Zhao et al. and the results were in a good range of adsorption ratio [24]. Furthermore, Ribeiro et al. investigated the deposition of HA by the PVDF polymer process on commercially pure titanium surfaces and the evaluation was registered by a modified laser beam irradiation [9]. In another study, Alexandre et al. characterized the PVDF/HAP composites for medical applications [25]. Ohtsuki et al. reported the evaluation of the bioactivity of HA nuclei with PVDF composites in a solid film mode [26]. Taking into account all studies on PVDF/HA composites, the PVDF coating on the surface of the composite was not conducive in the coating process. Therefore, finding ways to improve the hydrophilicity and electrical feature of PVDF coating is an obstacle in the application of piezoelectric PVDF. 

In this study, PVDF combination offers great potential for composite development due to the stability of HA, as silver ions can improve durability, bioactivity, and electrical properties. Therefore, samples consisting of a mixture of PVDF and HA in two different percentages of HA and PVDF, HA, and AgNO_3_ were prepared and the characterization, hydrophilic and electrical properties were discussed in detail. Thus, designed novel composites with obtained exceptional properties may be applied in such application areas as energy harvesting, self-powered sensors, wearable or portable electronics, etc. 

## 2. Experimental Methods

### 2.1. Materials and Instruments

All chemical reagents and solvents were analytical grade, supplied by Merck Co., Rahway, NJ, USA. In addition, X-ray and phase series were confirmed by X-ray diffraction (XRD) and performed on a Philips XRD diffractometer using Cu_kα_ radiation at 40 KV, 30 mA, a step size of 0.05° (2ϴ), and a scan rate of 1°/min. Fourier transform infrared spectroscopy (FTIR) of the samples was performed by the instrument Perkin–Elmer spectrometer BX FT-IR. Moreover, a scanning electron microscopy analysis (SEM) Phillips/FEI Quanta 200 was used to study the morphology of the samples. Furthermore, an energy-dispersive X-ray spectrometer (EDX) Phillips/ FEI 149 Quanta 200 was used to study the chemical elements of the components. The PicoScope USB oscilloscope system was chosen to study the electrical properties, and steel/Samples/Aluminum devices were fabricated. Since 1 millivolt [mV] = 0.001 watt/ampere [W/A], the values extracted from the piezoelectric measurement were registered as W/A. In addition, a millivolt (mV) is a decimal fraction and a watt per ampere (W/A) is equal to a volt (V), the unit derived from SI for electromotive force, electric potential (voltage), and electric potential difference. In addition, the hydrophilicity of spin-coated samples on the solid films was studied using doped water and double convex lenses, and the Guppy F-503 B&W CMOS camera. The devices were fabricated (steel/specimens/aluminum) and the physical momentum was done. 

### 2.2. Synthesis of HA

**Figure 1:**
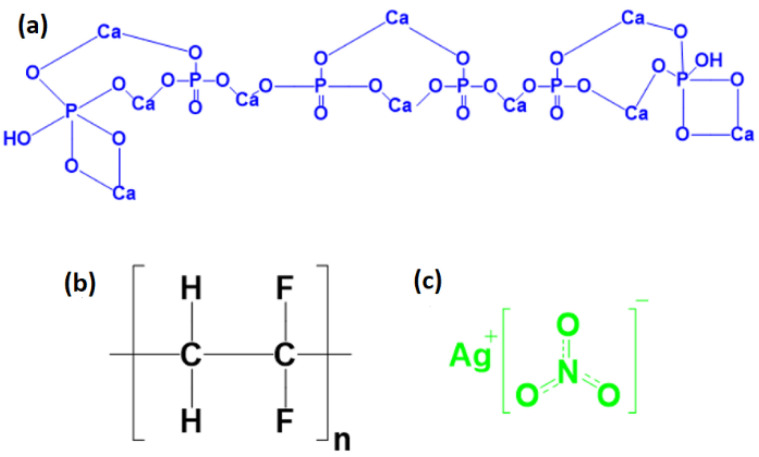
The chemical structures of compositions (**a**) HA, (**b**) PVDF, and (**c**) AgNO_3_.

The chemical structures of PVDF, HA, and AgNO_3_ are shown in Figure 1.

**Figure 2:**
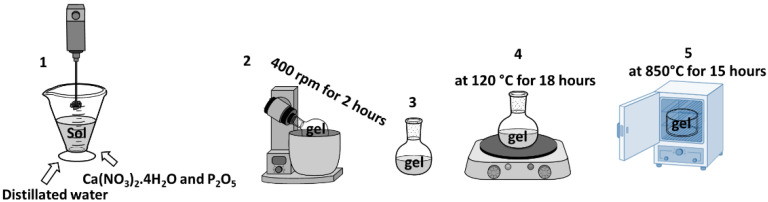
The synthesis route (Sol-gel method) of HA.

In this study, the pathway for the synthesis of artificial HA is shown in Figure 2. Calcium nitrate tetrahydrate (Ca(NO_3_)_2_.4H_2_O) and phosphorus pentoxide (P_2_O_5_) were used as precursors in a 10:3 molar ratio. (1) Ca(NO_3_)_2_.4H_2_O and P_2_O_5_ were dissolved in 10 mL ethyl alcohol (C_2_H_5_OH) and distilled water. (2) The product was stirred at 400 rpm for 2 h. (3) The gel was prepared at the bottom of the dish. (4) The gel was then air dried at 120 °C for 20 h. (5) For sintering, heat treatment was performed at 850 °C for 15 h. A similar procedure is described in Ref [27,28].

### 2.3. Preparation of Composites

**Figure 3:**
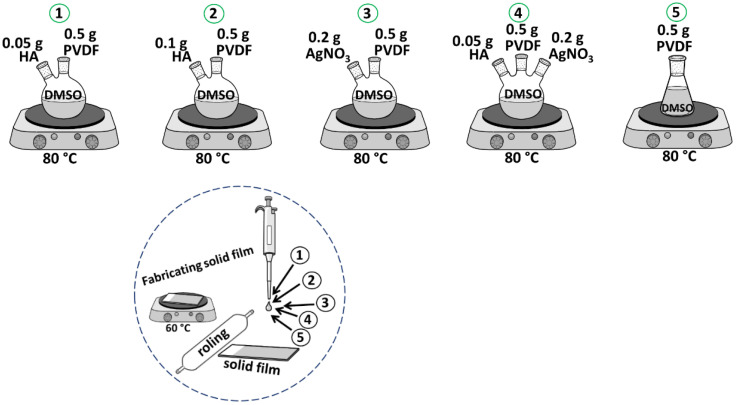
Schematic of synthesis route and fabrication of composites solid films.

The schematic routs for sample preparation are shown in Figure 3. In addition, the precursor values are listed in Table 1. 

Taking into account the initial characteristic of HA, PVDF, and AgNO_3_, four categories of new composites involved five samples consisting of 0.05 g HA:0.5 g PVDF (1), 0.1 g HA:0.5 g PVDF (2), 0.2 g AgNO_3_:0.5 g PVDF (3), 0.5 g HA:0.5 g PVDF:0.2 g AgNO_3_ (4), and 0.5 g PVDF (5) were prepared at 80 °C for 5 h till colloidal phase. In the next step, 200 µL of the liquid samples were applied to fabricate the films, and then the thin layer was rolled and dried at 60 °C. DMSO played a good role and heat treatment was performed at 80 °C due to the ice phase of DMSO at room temperature. In the four categories of prepared composites (1, 2, 3, 4), PVDF played the role of a matrix, HA and AgNO_3_ played the role of a reinforcement. In fact, DMSO has played a role as a solvent because it is an alternative solvent with low toxicity [29,30]. DMSO has low toxicity and is a non- hazardous solvent capable of dissolving high concentrations of PVDF at room temperature or mild temperatures [30]. The use of DMSO for the preparation of composites has been extensively documented in the literature. For example, Marino et al. [29], prepared a composite based on polyethersulfone (PES) by phase separation using a pleasant-smelling version of DMSO. Moreover, Enayatzadeh et al. [31] and Arefi–Oskoui et al. [32], reported thermodynamic and kinetic studies and the influence of different variables amount of PVDF on the physicochemical properties of the composite.

## 3. Results and Discussion

### 3.1. Study of X-ray Diffraction

**Figure 4:**
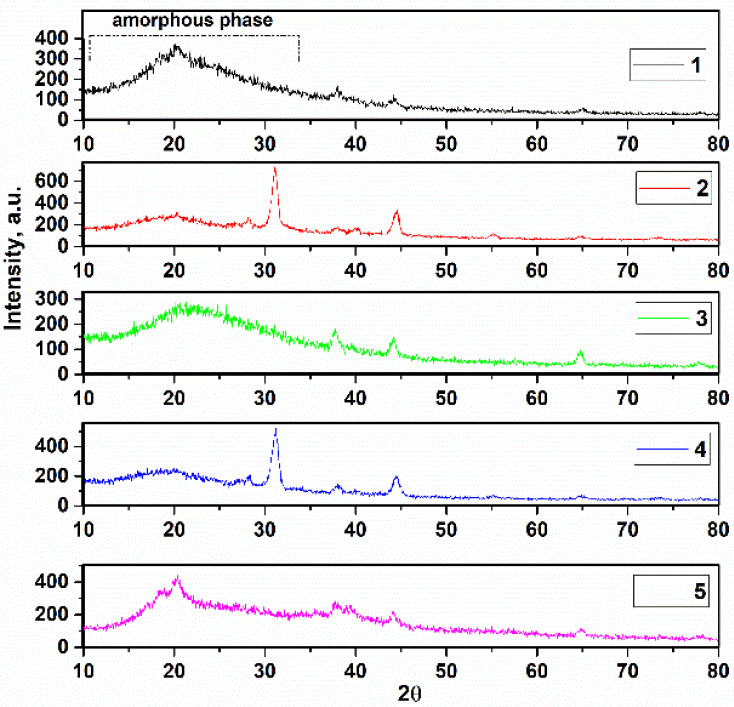
X-ray diffraction of deposited thin films.

X-ray diffraction of the samples in solid film mode was performed in the 2θ range from 10° to 80°, shown in Figure 4. It is noteworthy that complete crystallization was not observed in samples 1, 3, and 5, and these samples exhibit mixed amorphous and crystallite phases. This can be due to the PVDF content and the low temperature during heat treatment (80 °C) [33,34]. There are two phases of PVDF, α and β. The α-phase is cited with the monoclinic symmetry P21/c group and the β-phase with the orthorhombic symmetry Cm2m space group [35]. The sharp broad main peaks at 2θ = 19.89°, 21.20°, and 20.38° for these samples are assigned to (110), confirming that the original β-phase of PVDF was used in this study [36]. Furthermore, the α-phase (020) can be monoclinic PVDF due to the amorphous phase and overlap with the α-phase (020), as this interpretation is announced in ref [37]. This is attributed to the reaction between the solute and solvent [38]. There is no extreme effect of annealing temperature on the thin film when the XRD patterns corresponded to previous research such as Satapathy et al., who used the solvent DMSO and obtained a maximum percentage of β-phase in PVDF thin films when annealed at 90 °C for 5 h [39]. Or Imtiaz Noor Bhatti et al. obtained a similar result to Satapathy using acetone as a solvent, but pointed out that 2θ = 20.3° belong to the β-phase [40]. The absence of the crystalline phase was caused to dipolar interactions between C=O and CH_2_-CF_2_ in DMSO and PVDF [41]. The weak peaks at about 37.92° and 44.05° are attributed to the β-phase of PVDF in samples 1, 3, and 5, respectively. In addition, samples 2 and 4 showed a mixed phase of amorphous and crystalline, which can be attributed to the use of HA as a reinforcement. This can be due to weak hydrogen bonds C=O....H-C, both of which interfere with the interchain forces of PVDF:HA when the phases are mixed [42]. The sharp peaks at 2θ = 31.15° and 31.23° are attributed to (211) of HA, confirming the evidence of HA. Due to the existence of the amorphous phase in all XRD patterns and the peak positions of AgNO_3_ between 20° and 25°, there are two peaks in this region; therefore, the existence of the peaks of AgNO_3_ is due to the overlap of this amorphous region. Nevertheless, the existence of AgNO_3_ can be investigated by quantities X-ray Edax analysis. 

### 3.2. Study of FTIR

**Figure 5:**
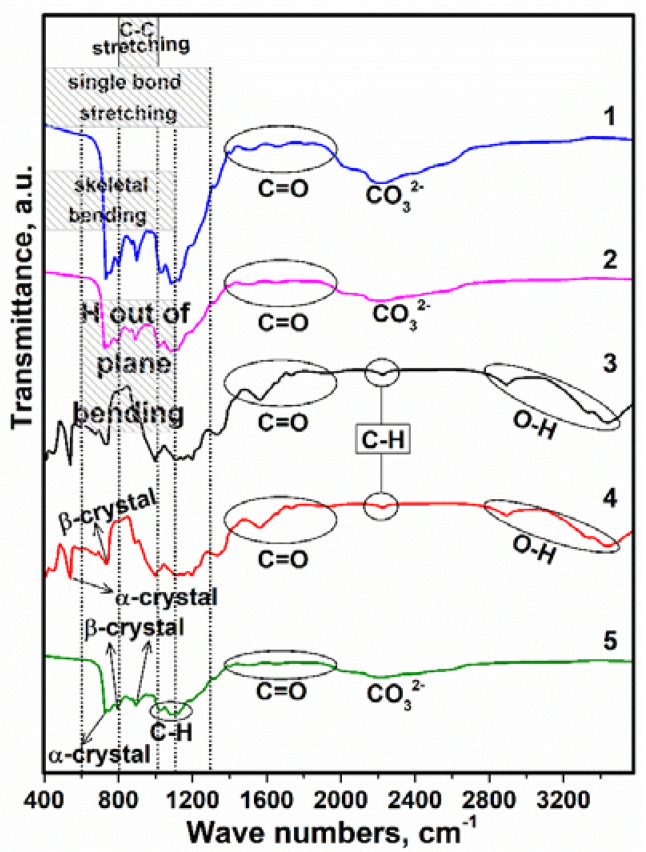
FTIR spectrum of deposited thin films.

The next step in the research was an analysis of FTIR spectra of the samples, given in Figure 5. According to these spectra, there was a greater presence of bands related to the α–and β–phase of PVDF, especially at 537, 724, 734, and 895 cm^−1^ [43,44]. The FTIR spectrum of samples 1, 2, and 5 are in strong agreement with each other. There are slight differences in the percent transmittance, which is higher for sample 1 compared to 2 and 5 due to the PVDF content. Taking into account the amorphous phases of the samples, the major base compositions of HA such as ${\mathrm{PO}}_{4}^{3-}$ can overlap with the α– and β–phase of PVDF, as the effects of the presence of ${\mathrm{PO}}_{4}^{3-}$ are in the 731, 1024, and 1088 cm^−1^ vibrational modes [45,46]. There was, also, a significant decrease in the intensity of the bands in the ${\mathrm{PO}}_{4}^{3-}$ region derived from HA, wherein the α–phase decreased significantly when the spectrum of PVDF was compared with the spectra of pure HA. The band at 1193 cm^−1^ (β–phase of PVDF) contributed to the increase in the intensity of the band at 1046 cm^−1^, which should have already been present in the spectrum of the composition HA. The spectrum is attributed to bonds between C and O in all samples in the range from 1375 to 1982 cm^−1^ [47]. In addition, the stretch band at 1340 cm^−1^ can be due to the presence of C–F bond [38]. Moreover, the HA constituents, –OH groups have indicated the existence of PVDF, and the wide range of wavenumber value from 2787 to 3548 cm^−1^ can be caused [48]. The impressive effect of the existence of HA and AgNO_3_ was not observed in the FTIR spectrum, because the content of this composition and also the value of the crystallization temperature was not high (80 °C), while the sintering and crystallization temperatures of HA and AgNO_3_ were more than ~500 °C [49]. Consequently, the FTIR conformed to the XRD results to transfer the phase and ingredients of the composites.

### 3.3. SEM Analysis

**Figure 6:**
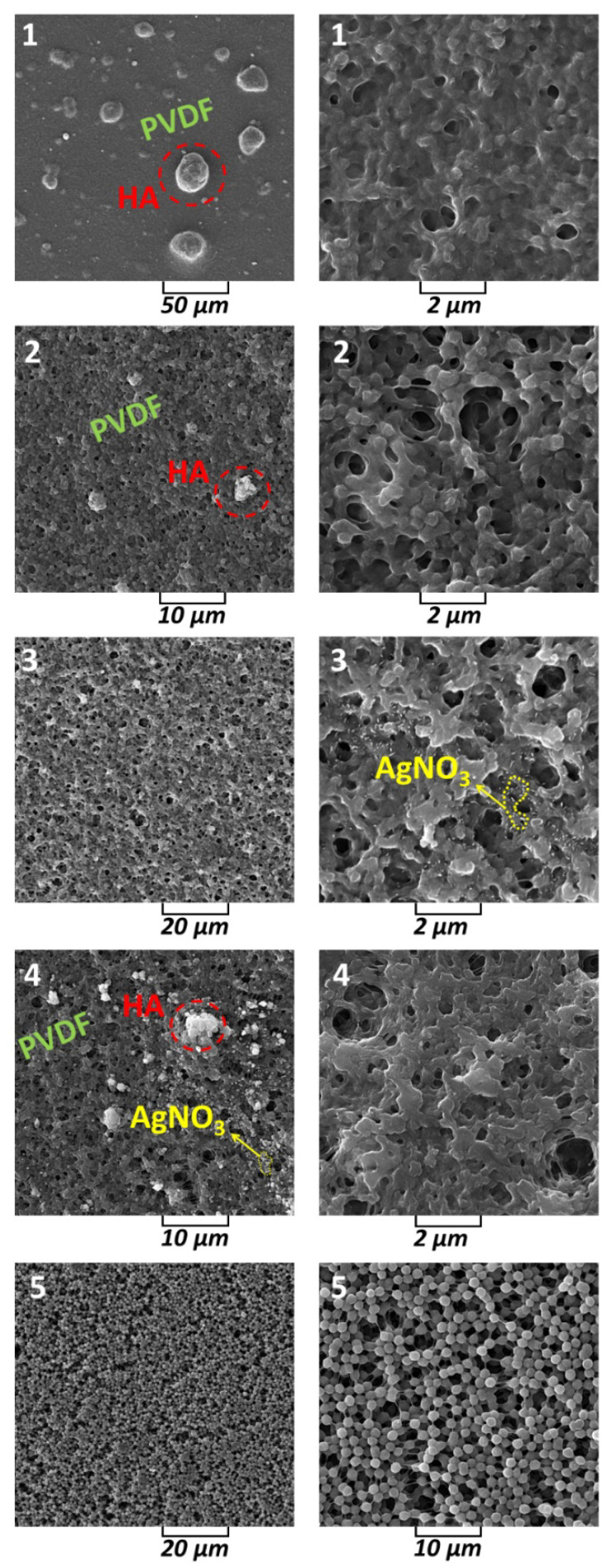
SEM images of samples 1, 2, 3, 4, and 5 at two different magnifications.

Morphological examination of the samples was performed using SEM and obtained 3D views are given in Figure 6. Thus, in Figure 6 for sample 1, it is clear that the PVDF is the matrix and the nucleation, and the growth of HA on the PVDF occurs simultaneously. Due to the amorphous phases, the interpretation of the size of the nanocrystallites by X-ray diffraction was not possible, but in the SEM image for sample 1 and 2, the spherical HA with a diameter of ~25 and 5 µm, respectively, can be predicted compared to the pure PVDF. It is worth mentioning that by Cotica et al. HA:PVDF composite was prepared based on an electrospinning process and the HA particles were nucleated on the PVDF, and HA played a role as reinforcement [50]. Furthermore, the similarity of the images of samples 1 and 2 is related to the similar composition of these composites. For sample 3, it is noted that the incorporation of AgNO_3_ was satisfactory with good dispersion of these particles in the skeleton, which was due to the use of a small amount of AgNO_3_ in the preparation of sample 3. The presence of all three components of the composites PVDF, HA and AgNO_3_ led to a reduction in the size of the nucleated particles in sample 4. This phenomenon was also observed by Tandon et al. for composites with PVDF as the main component [51]. These slight differences between samples can be related to the use of DMSO as a solvent and the evaporation rate, which may increase viscosity and change conductivity. The uniform structure is shown in the SEM image for sample 5 (PVDF only). The strand morphology of PVDF (Figure 6, sample 5) was observed when the irregular porosities were done in the composites. The reasons for this can be related to the properties of the used solvent (DMSO), especially, the polarity features and the applied low temperature. This is because DMSO is a polar aprotic solvent that dissolves both polar and nonpolar compounds, and is miscible with a wide range of organic solvents. In this case, the polarity of DMSO caused the amorphous phases and the strong dipoles of the C–F bond of the molecular chain in the PVDF to rotate and reduce the energy barrier for the formation of the extended trans-conformation [52,53]. Thus, the high molecular weight of the composites can prevent the PVDF from flowing freely on the surface during the thermal activation process. 

In this research, the creation of an amorphous phase was advantageous because the PVDF has a crystalline form and the molecular chains do not have a fixed structure and can move freely, which negatively affects the electrical properties [54]. Taking into account X-ray diffraction and SEM images, it is understandable that when low temperatures (80 °C) are used the reaction energy between the PVDF molecular chains is greater than the reaction energy of the interaction of PVDF:DMSO; therefore, the crystalline region of PVDF remains virtually passive and swelling occurs as the solvent penetrates the amorphous region. Thus, the traces of evaporated DMSO are the pores seen in the SEM images of samples 1, 2, 3, and 4. As evidence for this interpretation, Chinaglia et al. found that low temperatures favor the formation of the β–phase in PVDF [55]. It is significant that the crystallization process of PVDF can start with the nucleation in several steps, the formation of liquid-like clusters, and the rate-limiting organization of such clusters into crystal nuclei. Therefore, according to the XRD patterns and morphology analysis via SEM, high temperature was not used and time was not given for crystallization of PVDF or/and HA:AgNO_3_ [56].

### 3.4. Investigation of EDX Analysis

**Figure 7:**
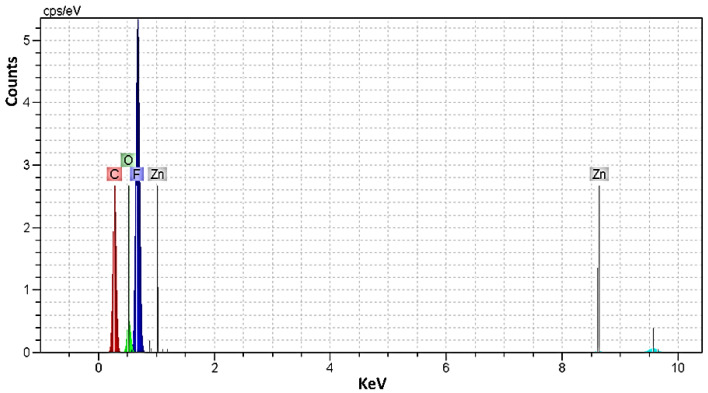
The EDX curve of sample 5 (PVDF) as the main composition.

EDX analysis was used to identify the chemical elements and their concentration in the precursors. The EDX spectra of PVDF (sample 5, Figure 7) [57] and the semi-quantitative ratio of chemical elements were calculated based on the peak spectrum, and the obtained values were tabulated in Table 2. In addition, in this research, the zinc (Zn) was chosen as the resource of the instrument and the values of Table 2 confirmed that there are no impurities in the composite structure. The homogeneous distribution of the elements can be seen in the extracted values of the EDX analysis. Moreover, the presence of chemical elements such as C, O, F, Ca, Ag, and P is detected in the EDX analysis, which clearly indicates the successful preparation of the samples. The small differences between the values confirm the considered presence of the elements in the preparation of composites. As expected, the fluorine peak originating from PVDF is particularly intense, especially in PVDF (sample 5). 

Taking into account Table 2, when HA is used as reinforcement, the weight fraction of fluorine decreased to 47.37% and the composition of the main elements of HA had a ratio of $\frac{\mathrm{Ca}}{P}$ = 1.71 for sample 1 and $\frac{\mathrm{Ca}}{P}$ = 1.66 for sample 2, respectively. The presence of AgNO_3_ as a component of the re-reinforcement was detected in composite 3. Furthermore, in sample 4, the addition of AgNO_3_ as an additional reinforcement reduced the contents of Ca and P, but the structure of HA was maintained, since the ratio of $\frac{\mathrm{Ca}}{P}$ = 1.68 and these values were in good agreement with natural HA ($\frac{\mathrm{Ca}}{P}$ = 1.67) [28,58,59]. Additionally, the values of samples 1, 2, and 4 showed the growth HA on the surfaces of PVDF with good distribution. Moreover, the elemental analysis showed a significant increase in the percentage of C atoms in sample 5 compared to the composites.

### 3.5. Study of Electrical Potential

**Figure 8:**
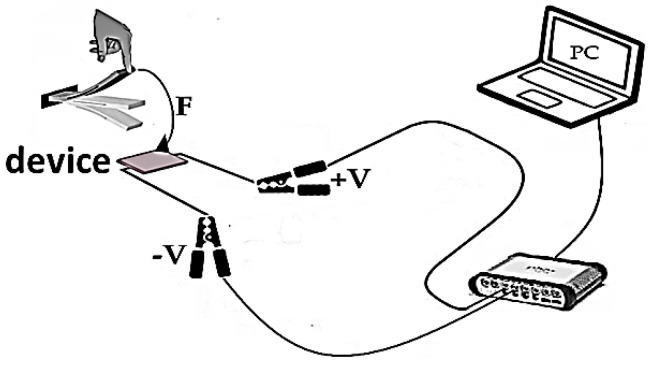
Schematic routes for the evaluation of the electrical properties of the devices.

Five devices based on specimens were fabricated to study the electrical properties (samples 1, 2, 3, 4, and 5). The devices consisted of three layers—steel/specimens/aluminum. Moreover, the voltage was measured in an open circuit and a similar investigation was carried out in the reference [60]. The schematic route of the investigation electrical properties of the devices is shown in Figure 8.

The values extracted from the electrical investigation are listed in Table 3. The difference between the values is related to the active layer, which samples 3 and 5 do not have. The device that consisted of sample 2 as the active layer showed the highest amount of energy ~0.12 ± 0.01 W/A. It can be seen that HA was suitable as a reinforcement for increasing the electrical durability, since the devices that consisted of samples 3 and 5 (without HA) showed the lowest energy values. The reason for this lies in the semi-empirical, automatically estimated piezoelectric constants that can be obtained in the crystal structure of HA and the initial property that HA can exhibit piezoelectric properties [61,62]. It is worth noting that the creation of ferroelectric properties by PVDF can also increase the electric charge barrier and the mobility at the room temperature [63].

### 3.6. Study of Hydrophilicity

**Figure 9:**
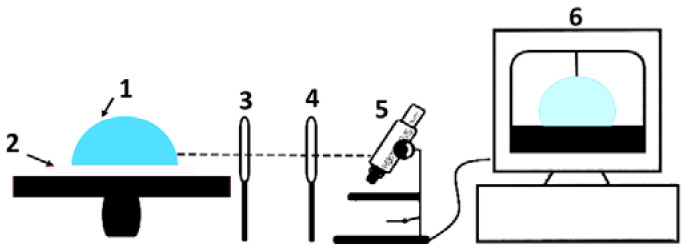
Hydrophilicity measurement; (1) drop on the specimens, (2) substrate of coating, (3,4) convex double lenses, (5) Guppy CMOS camera for detecting, and (6) software system.

Contact angle measurements were performed to evaluate the wettability and surface free energy of the samples. The schematic routes for measuring the angles are illustrated in Figure 9. Knowledge of the wetting behavior of solvents on solid surfaces is important in order to study the intermolecular communication between the piezoelectric solid surface and the liquid-analyte, and to improve the electrical properties of the prepared samples. The intensity of wetting determines the balance of forces between the adhesion and cohesion [64]. 

**Figure 10:**
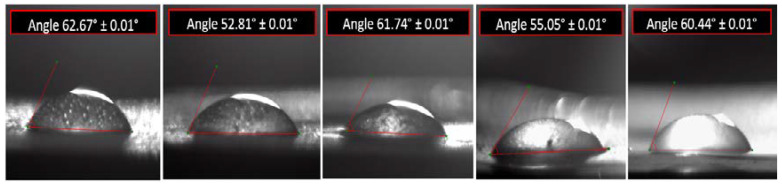
Droplet images and angles of wettability of coated samples on the solid film mode.

Figure 10 shows the average angles between a drop of water and spin-coated samples on the solid film. The average angle values (θ_Young_) ranged from 52.81° to 62.67° and the lowest angle belonged to spin-coated sample 2 on the film. The good wetting can be reported based on θ < 90° for all samples [65]. It is proved that sample 2 has a better agreement with the liquid than the other samples and has the highest surface energy [66,67]. The result indicates that the surface of spin-coated sample 2 has the highest polarity and the highest uniformity on the solid film.

Overall, some close previous studies and performances are brought in Table 4, as a short comparison.

## 4. Conclusions

In this study, the artificial HA was synthesized by the sol-gel method. The samples consisting of three compositions PVDF, HA, and AgNO_3_ were successfully prepared by a facile casting method. In this process, HA and AgNO_3_ played the role of the reinforcement and PVDF played the role of the matrix. The X-ray diffraction of the spin-coated samples on the solid films showed the amorphous and mixed amorphous with crystalline phases. The FTIR spectrum of the samples proved the bonding between the constituents of the samples. In addition, the SEM images showed and conformed to the amorphous phases of the samples. The EDX analysis determined that there are no impurities in the composition of the sample structures and that the content of the compositions was consistent with the precursors. The $\frac{\mathrm{Ca}}{P}$ ratio proved the existence of HA in the production of the composites. The electrical properties were studied and the highest extracted energy values of the devices, composed of PVDF/HA/AgNO_3_ layers, were registered in the range from 56.50 to 125.20 mV. In addition, the hydrophilicity of the samples was investigated and θ_Young_ was determined in the range from 52.81° to 62.67°, and was the lowest value belonging to sample 2. This sample exhibited the highest polarity and surface energy, too.

Overall, HA performed well in this series of composites and the presence of HA increased the electrical properties, polarity, and uniformity of the composites, based on PVDF due to the initial piezoelectric property of HA. Thus, the obtained results imply that designed composites may be identified as potential materials for energy-harvesting applications.

## Figures and Tables

**Table 1:** Values of precursor for preparing samples.

Sample	PVDF (g)	HA (g)	AgNO_3_ (g)
(1)	0.5	0.05	-
(2)	0.5	0.10	-
(3)	0.5	-	0.2
(4)	0.5	0.05	0.2
(5)	0.5	-	-

**Table 2:** The extracted stoichiometric values from EDX analysis of specimens.

Element	1	2	3	4	5
	Weight, %				
**Carbon**	17.37	19.16	17.04	15.04	22.01
**Oxygen**	5.30	5.18	8.53	8.73	6.29
**Fluorine**	53.01	47.37	65.61	47.60	69.54
**Calcium**	13.91	16.41	-	12.94	-
**Silver**	-	-	6.79	6.01	-
**Phosphorus**	8.09	9.83	-	7.70	-
**Zinc**	2.32	2.05	2.03	1.98	2.16

**Table 3:** The extracted energy values from electrical properties of devices.

Sample					
**Energy (W/A)**	**1**	**2**	**3**	**4**	**5**
	0.08 ± 0.01	0.12 ± 0.01	0.06 ± 0.01	0.09 ± 0.01	0.05 ± 0.01

**Table 4:** Brief comparison of some previous studies on the subject of PVDF composites.

Sample	Short Explanation	Ref.
**PVDF/HA (60:40)**	The stress was reported 459.2 ± 4.1 MPa, and deformation was 0.23 mm	[4]
**PVDF/NaY** **zeolite composites**	Solvent such as DMSO and DMF were compared and the important difference was related to dielectric constant	[68]
**PVDF in DMSO solvent**	The phase characterization was done and maximum β-phase was appeared in the films when PVDF was annealed at 90 °C for 5 h	[39]
**PVDF-BaTiO_3_-Ag**	Fibers showed an increase in the output voltage (1.78 (12) mV) compared to pristine PVDF and PVDF-BaTiO_3_ composite fibers (1.48(26) mV) upon applying 1.2 N force at 5 Hz frequency	[69]
**PVDF in DMSO solvent**	PVDF films with high content of β-phase up to 98.8% was obtained by using DMSO as the solvent at optimized crystallizing temperature of 60 °C	[17]
**HA/PVDF**	The HA content from [0% to 15%] reduced the d33 constant from 2.61 pC/N to 1.08 pC/N, while the HA content increased further to 20%, the d33 value increased to 1.53 pC/N. The addition of HA changed the amount of β-PVDF, which determined the piezoelectric performance of the HA/PVDF composite	[3]
**PVA/AgNO_3_**	The high antibacterial activity was found to series of the samples dried at 25 °C. The antibacterial activity of the investigated samples was ascribed to silver ions formed during the samples dissolution in the presence of water	[70]
**PVDF/HA/AgNO_3_**	This study	

## Data Availability

Data sharing is not applicable.
